# Changes in Sperm Parameters with Time in Men with Normal and Abnormal Baseline Semen Analysis

**DOI:** 10.1007/s43032-024-01475-1

**Published:** 2024-02-29

**Authors:** Nir Cohen, Assaf Ben-Meir, Tzuf Harlap, Tal Imbar, Gilad Karavani

**Affiliations:** 1 Infertility and IVF Unit, Department of Obstetrics and Gynecology, Hadassah Ein-Kerem Medical Center and Faculty of Medicine, Hebrew University of Jerusalem, Jerusalem, Israel; 2 Department of Obstetrics and Gynecology, Hadassah Ein-Kerem Medical Center and Faculty of Medicine, Hebrew University of Jerusalem, Jerusalem, Israel; 3 Faculty of Medicine, Hebrew University of Jerusalem, Jerusalem, Israel; 4 Division of Urology, Department of Surgery, Mount Sinai Hospital and New Women’s College Hospital, University of Toronto, Toronto, ON Canada

**Keywords:** Male infertility, Semen analysis, Age, Sperm quality

## Abstract

The association between paternal age and sperm quality in the population level has been previously studied. Only limited data exists regarding the intra-personal variations in semen parameters among fertile and infertile men over time. We aimed to assess trends over time in semen parameters among men with normal and abnormal baseline sperm parameters and investigate potential risk factors for sperm quality deterioration. This retrospective cohort study was conducted at a university-affiliated medical center in vitro fertilization (IVF) unit. Patients with at least two semen analyses (SA) performed > 1 year apart, with the last SA done between 2017 and 2021, were included. The study consisted of two main analyses—comparison of intra-patient’s sperm parameters changes in men with normal and abnormal baseline SA (BSA) and analysis of risk factors for developing abnormal semen parameters over time in men who had normal BSA parameters. This study included a total of 902 men assessed for infertility with normal and abnormal BSA. The average time interval between tests was 1015 days (range 366–7709 days). Among individuals with normal BSA, there was a mild decline in most parameters—concentration (− 6.53 M/ml), motility (− 7.74%), and total motile count (TMC) (− 21.80 M) (*p* < 0.05 for all parameters). In contrast, a slight improvement in most parameters, except for concentration, was noted in men with abnormal BSA—volume (+ 0.21 ml), motility (+ 8.72%), and TMC (+ 14.38 M) (*p* < 0.05 for all parameters). Focusing on men with normal BSA, 33.5% of individuals developed abnormality in one or more of their sperm parameters over time, within a mean time of 1013 ± 661 days. We also found that only time between tests emerged as an independent prognostic factor for the development of abnormal SA later. Interestingly, sperm deterioration in participants in their third, fourth, and fifth decades of life with normal initial semen analysis was similar. Our study provides evidence of a decline in semen quality over time in individuals with normal BSA, in contrast to men with abnormal BSA. Longer time intervals between tests independently increase the risk of sperm abnormalities.

**Supplementary Information:**

The online version contains supplementary material available at 10.1007/s43032-024-01475-1.

## Introduction

Sperm count, a crucial measure of male fertility, has been a topic of growing interest and concern in recent years. Male factor infertility plays a significant role in the challenges faced by couples trying to conceive, accounting for nearly half of infertility cases and solely responsible for 20–30% of them [1, 2]. Globally, approximately one in ten men experience infertility or subfertility. This condition can be attributed to various causes, including genetic conditions that lead to reduced sperm concentration (oligospermia) or complete absence of sperm (azoospermia). Other factors, such as endocrine-disrupting chemicals, smoking [3], body mass index (BMI) [4, 5], geographical location [6, 7], and paternal age [8], have also been found to affect semen quality parameters such as semen volume, sperm concentration, and degree of sperm motility.

Previous studies have provided valuable insights into semen quality trends, highlighting a potential temporal decline in sperm count. Notably, a study by Carlsen et al. [9] reported a substantial decrease in sperm concentration in previous decades, followed by subsequent investigations of the trajectory of sperm count changes, yielding important insights. For instance, a meta-regression study by Levine et al. (2017) [6, 7] analyzed semen samples from 42,935 men between 1973 and 2011, revealing an overall decline of 52.4% in sperm concentration and 59.3% in total sperm count over the past 40 years. However, it is important to acknowledge that these and other studies have focused mostly on sperm parameter changes across generational cohorts over extended periods [10, 11]. Moreover, those studies have some limitations, such as variations in laboratory testing methods, technological advancements, and the consideration of patient-specific factors (such as health status, fertility status, and ethnicity). Furthermore, while population-level trends have been extensively studied, changes over time in the individual level have received less attention. Consequently, there remains a need for large-scale studies examining intra-individual changes in sperm quality, count, and concentration, longitudinally in men over clinically significant periods of time.

To address these gaps, our study aimed to track changes in semen parameters in the individual level over time among men with initial normal and abnormal semen parameters undergoing in vitro fertilization (IVF) treatments, who have provided at least two semen analyses (SA) over a minimum 1-year interval.

## Methods

### Study Population

This was a retrospective cohort study which included data from a university-affiliated infertility and IVF unit. We reviewed data of men who attended our IVF clinic as part of the evaluation of the infertile couple between 2017 and 2022. Collected data included demographic information and general medical background, lifestyle-related data, and prior reproductive related data. Men included in this study had at least two documented semen analysis reports, done 1 year or more apart in an andrology laboratory. Men with an initial SA analysis that showed azoospermia (determined by a semen sample in which sperm was not observed in the replicate wet preparations followed by centrifugation at 3000 g (for approximately 15 min)) without spermatozoa found were excluded from the study as well as those who had only one SA report or that their last semen analysis was done within a year from their documented baseline SA.

### Outcomes

The primary outcomes of this study were the intra-personal changes in sperm parameters (volume, concentration, motility, and TMC) between the baseline semen analyses (BSA) and the last documented SA (LSA). Secondary outcomes were risk factors associated with developing abnormal sperm parameters in those who had initial normal semen parameters.

The study population was divided into groups based on the normal or abnormal sperm definition derived from the BSA and in accordance with the World Health Organization (WHO) Manual for Human Semen Analysis 5th edition [12] guidelines. Men with one or more abnormal parameters in their baseline SA—volume (under 1.5 ml), concentration (under 15 million/ml (M/ml)), and motility (under 40% total motility or < 32% progressive motility)—were allocated to the abnormal BSA group, while those with all normal parameters were categorized as normal BSA. We compared the baseline characteristics, reproductive data, and sperm parameters between the groups. Additionally, we calculated the mean change per patient in each sperm parameter within each group.

An additional analysis specifically focused on the subgroup of individuals who initially had a normal BSA. Within this subgroup, we compared the basic characteristics and results of the baseline and last SAs between those who subsequently developed abnormal sperm parameters and those who maintained a normal SA in the last test.

Furthermore, we employed univariate and multivariate logistic regression models to predict the development of oligospermia over time in the entire study population and performed similar regression analyses within the subgroup of individuals with a normal BSA.

### Specimen Collection and Evaluation

Following 2–7 days of abstinence, men provided a semen sample by masturbation. Samples were evaluated in the andrology laboratory at Hadassah Medical Organization in Jerusalem or at another hospital or community-registered andrology laboratory in the country. Semen was analyzed according to the 2010 WHO criteria [12] for volume (milliliter (ml)), concentration (million/ml), motility (%), normal morphology(%), and TMC—total number of motile sperm in the entire sample (million (M)). The value of sperm concentration and motility were evaluated by Makler chamber and/or computer-assisted sperm analysis (CASA). Sperm morphology assessment was done by microscopic evaluation of sperm structural characteristics.

### Statistical Analysis

Categorical data was described as proportions and the significance between groups for quantitative parameters was assessed by the Chi-square test or Fisher’s exact test, as indicated. These variables were compared between two independent groups (normal and abnormal BSA) using the *t*-test or chi-square test, and intra-personal SA changes were assessed using paired *t*-test, as we compared only two semen analyses. Additionally, a logistic regression model was applied to search for independent predictors for the dichotomous-dependent parameter of a defined normal or abnormal LSA, reporting odds ratio (OR) for each parameter with a 95% confidence interval (CI) using univariate and multivariate logistic regression models. The Omnibus Tests of Model Coefficients and the Hosmer–Lemeshow test were used to test for goodness of fit for these models.

Sample size calculation was based on estimated annual sperm concentration decrease rates of 3.9% [13] and 11.7% overall in 3 years (average time between tests in our study) with a standard deviation estimation of 40. This calculation showed that a selection of a random sample of 828 patients with the determination that the mean of the differences is 7.0, and the standard deviation of the differences is 40, has 80% power to declare that the mean of the paired differences is significantly different from 0.

A *p*-value of < 0.05 was considered statistically significant for all comparisons, with all tests being two-tailed. We used the 4.0.0 version of the R Foundation for Statistical Computing for the statistical analysis.

### Ethical Approval

This study conforms to the provisions of the declaration of Helsinki and was approved by the Human Research Ethics Committees of the Hadassah University hospital (IRB 0778–21-HMO).

## Results

A total of 3567 men underwent evaluation at our IVF unit, with at least one SA reported during the study period. Of them, we excluded 1581 men with only one SA recorded, and 1039 men had their LSA done within a year from their BSA were excluded, and of the remaining 947 men, we excluded additional 45 men who had a BSA which showed azoospermia.

Finally, 902 men who had two semen analysis results done with a minimum interval of 1 year, between 2017 and 2021, and a BSA which showed the presence of sperm in the ejaculate were included in the final analysis. The average age at baseline SA was 37.6 ± 7.8 years, and the average time between tests was 1015 ± 739 days (range 366–7709 days). A comparison of basic characteristics and initial SA sperm parameters was done between the abnormal and normal BSA groups (Table 1). Significant differences were observed between the groups in terms of surgical background (*p* = 0.03), with higher proportion of urological surgeries in the abnormal BSA group (7.4% vs. 2.7%) and, as expected, poorer sperm parameters at BSA in the abnormal BSA group compared to the normal BSA group (Table 1).

**Table 1:** Basic characteristics and baseline semen analysis data of the study population at first visit to the infertility clinic

Parameter	Normal BSA	Abnormal BSA	*p*-value
No. of patients	334	568	
Age(mean)	38.1 ± 7.6	37.3 ± 8.1	0.158
Time between SA (days)	1012 ± 662	1018 ± 782	0.915
Smoker	36 (10.8%)	56 (10%)	0.660
Alcohol	0	4 (0.7%)	0.124
Drugs			0.749
No treatment	308 (92.5%)	515 (90.5%)	
Previous hormonal	0	4 (1%)	
Cardiac or metabolic	11 (3.3%)	18 (3.2%)	
Other	14 (4.2%)	32 (5.6%)	
Medical history			0.403
None	313 (94%)	529 (93%)	
Chemotherapy	3 (0.9%)	3 (0.5%)	
Urologic diseases	0	5 (1%)	
Chronic disease	17(5.1%)	31 (5.4%)	
Obesity (BMI > 35)	0	1 (0.2%)	
Testicular injury	0	2 (0.4%)	
Surgical background			0.03
No previous surgery	284 (85%)	458 (80%)	
Urologic	9 (2.7%)	42 (7.4%)	
Other abdominal or pelvic	20 (6%)	32 (5.6%)	
Other	20 (6%)	37 (6.5%)	
Baseline semen parameters			
Volume (ml)	3.1 ± 3.1	2.8 ± 1.7	0.029
Concentration (M/ml)	69.5 ± 44.0	32.8 ± 50.7	< 0.001
Motility (%)	56.0 ± 12.6	21.7 ± 18.0	< 0.001
Sperm count (million)	200.0 ± 168.4	88.4 ± 151.7	< 0.001
TMC (million)	113.8 ± 96.9	23.9 ± 45.6	< 0.001

Data presented as mean ± standard deviation (*SD*) or *n* (%)

*SA*, semen analysis; *BSA*, baseline semen analysis; *TMC*, total motile count

Average intra-personal changes in each semen parameter between the baseline and last SA were calculated separately for the normal and abnormal BSA groups (Table 2). Men with normal BSA demonstrated a significant decrease of 6.8 ± 51.6 M/ml in sperm concentration (*p* = 0.022), 7.7 ± 23.2% in sperm motility (*p* < 0.005), and a 21.8 ± 123.5 million decrease in TMC (*p* = 0.007) between baseline and last SA. Conversely, sperm parameters did not deteriorate in the group of men with initial abnormal BSA, demonstrating a slight increase in volume (0.2 ml, *p* = 0.019), motility (8.7%, *p* < 0.005), and TMC (14.4 M, *p* < 0.005).

**Table 2:** Intra-personal changes in sperm parameters between baseline and last semen analyses in men with initial normal and abnormal sperm parameters

Parameter	Normal BSA		Abnormal BSA	
	Mean ± SD	*p*-value	Mean ± SD	*p*-value
Change in volume (ml)	− 0.2 ± 3.1	0.224	0.2 ± 2.0	0.019
Change in concentration (M/ml)	− 6.8 ± 51.6	0.022	− 0.9 ± 48.1	0.622
Change in motility (%)	− 7.7 ± 23.2	< 0.001	8.7 ± 22.0	< 0.001
Change in TMC (million)	− 21.8 ± 123.5	0.007	14.4 ± 66.1	< 0.001
Change in sperm count (million)	− 24.4 ± 205.1	0.038	2.4 ± 168.1	0.756

Data presented as mean ± standard deviation (*SD*)

*BSA*, baseline semen analysis; *TMC*, total motile count

Furthermore, an additional subgroup analysis was conducted to assess changes in sperm parameters among the normal BSA group, focusing on those whose one of their sperm parameters became abnormal (*n* = 112) and those who remained with completely normal LSA (*n* = 222). A comparison of characteristics between the normal (66.5%) and abnormal LSA (33.5%) showed significant differences in relation to time between tests, longer in the abnormal LSA group (1157 ± 778 vs. 938 ± 576 days, *p* < 0.005). Changes in semen parameters between normal LSA group and the abnormal LSA group were calculated separately for the normal BSA group (Suppl. Table 1). In the normal LSA group, no significant changes were found except for improvement in sperm motility (3.6%, *p* = 0.015). However, in the abnormal LSA group, there were significant decreases in all sperm parameters, except for semen volume. We observed an average decrease in concentration of 19.4 ± 46.3 M/ml (*p* < 0.005), a motility drop of 22.6 ± 21.3% (*p* < 0.005), and TMC which was lower by an average of 53.7 ± 67.6 million (*p* < 0.005). We then performed logistic regression models for predicting the deterioration to abnormal sperm in men with baseline normal sperm parameters (Table 3). This model, which included baseline characteristics and BSA data, did not identify predictors for this deterioration, except for time between BSA and LSA (continuous parameter (days with an OR = 1.01 (95%CI 1.00–1.01), *p* = 0.006; and categorical—3–5 years between tests—with an OR = 1.97 (95%CI 1.04–3.76), *p* = 0.038). No significance was observed in other covariates (age, smoking, medical and surgical background) including baseline sperm parameters.

**Table 3:** Multivariate logistic regression for the deterioration from normal baseline semen analysis to abnormal semen analysis with time

	OR (95%CI)	*p*-value
Age	1.003 (0.967, 1.041)	0.853
Days between baseline and last SA	1.001 (1.000, 1.001)	**0.006**
*Time between baseline and last SA (categorized)		
1–3 years	Referent	
3–5 years	1.975 (1.037, 3.763)	**0.038**
> 5 years	2.242 (0.955, 5.265)	0.064
Smoking	1.434 (0.623,3.3)	0.396
Medical background		
Past malignancy	2.532 (0.14, 45.651)	0.529
Chronic conditions	0.801 (0.199, 3.223)	0.754
Surgical background		
Urologic surgery	1.22 (0.279, 5.344)	0.792
Other abdominal or pelvic surgery	2.579 (0.903, 7.369)	0.077
Other	2.147 (0.738, 6.249)	0.161
Drug use		
Previous hormonal treatment	2.038 (0.39, 10.639)	0.399
Cardiac or metabolic	2.401 (0.682, 8.452)	0.173
Baseline semen parameters		
Volume (ml)	0.742 (0.526, 1.047)	0.090
Concentration (million/ml)	0.991 (0.977, 1.006)	0.230
Movement (%)	0.982 (0.943, 1.021)	0.358
Sperm count (million)	0.999 (0.989, 1.009)	0.826
TMC (million)	1.003 (0.987, 1.02)	0.689

*SA*, semen analysis; *TMC*, total motile count; *CI*, confidence interval

^*^Analyses performed using time as a categorical variable

Interestingly, age had no significant impact in our study. Average ages were similar between groups, 38.1 ± 7.6 for normal BSA and 37.3 ± 8.1 for abnormal BSA (*p* = 0.158). Within the normal BSA group, age differences between normal and abnormal LSA were also insignificant (38.1 ± 7.4 vs. 38.3 ± 7.9, *p* = 0.814). When assessing the predictive model for abnormal sperm count, age did not play a significant role (*p* = 0.853, OR = 1.003, 95% CI, 0.967–1.041), and introducing a cutoff at 40 years of age also yielded no significant impact (*p* = 0.821). (Suppl. Figure 1).

## Discussion

This study aimed to investigate intra-individual changes in sperm parameters with time in individuals with baseline normal vs. abnormal SA. Our findings revealed an overall decline in sperm parameters among individuals with normal baseline SA, while a slight improvement was observed in men with abnormal baseline SA over time. Within the normal BSA group, we found that the sperm quality of 33.5% of individuals became abnormal with time, while no significant changes were observed in the remaining individuals.

When analyzing the risk factors for sperm parameters deterioration below the normal range according to the WHO guidelines in men with normal BSA, we found that among the risk factors evaluated, only the time interval between semen analyses is a significant predictor for deterioration in sperm parameters, while baseline SA parameters are not.

Numerous factors have been linked to the decline in spermatogenesis, including advanced paternal age, environmental exposures, and chronic inflammation and illness [14–17]. Meta-analyses investigating the impact of advanced paternal age on sperm parameters have yielded conflicting results regarding the decline in sperm quality with increasing age. However, despite the ongoing debate, most studies have observed a gradual age-related decrease in sperm volume, motility, and to some extent sperm concentration [17, 18].

Our findings align with these studies, demonstrating an overall and gradual decline in sperm parameters over time. The time intervals evaluated in our study reflect the individual-level effects of aging and the possible cumulative impact of detrimental exogenous and endogenous exposures, which gradually impair sperm quality. Therefore, the long-term deterioration observed in most sperm parameters likely represents the natural deterioration of sperm quality in men.

Interestingly, men with abnormal BSA showed improved results with time. We speculate that the slight improvement observed in several sperm parameters among individuals with abnormal BSA may be attributed to a multifaceted array of interventions and lifestyle modifications implemented during the study period. This encompasses not only general lifestyle adjustments but also the introduction of fertility supplements designed to optimize reproductive health and the potential influence of medical or surgical interventions that were recommended and implemented. The comprehensive nature of these interventions, ranging from holistic lifestyle changes to targeted medical interventions, underscores the complex and interconnected factors that could contribute to the observed positive shifts in sperm parameters among individuals initially categorized with abnormal BSA. For example, it is well described that combining diet and exercise for weight loss has positive effects on sperm parameters [19]. Obesity was previously shown to impact semen quality, particularly at the epididymal and prostate levels. At the post-testicular level, obesity may adversely affect sperm count, motility, volume, inflammatory components, and DNA integrity, rather than the spermatogenesis reflected by morphology. This aligns with findings of abnormally high spontaneous acrosome reactions in morbidly obese men, who show improvements after bariatric surgery [20], indicating post-testicular events.

However, due to the retrospective study design and long follow-up, this data was not available to explore and analyze.

Regarding the clinical significance of the decrease in sperm counts, a definitive answer remains elusive. While we observed a significant overall decline in most semen parameters among individuals with normal BSA, particularly those who developed abnormal sperm parameters between tests, it is important to note that surpassing the standardized reference limits for normal sperm parameters established by the WHO guidelines [12] does not solely account for male infertility. Rather, it is likely that one or more semen variables contribute to a multifactorial condition or disease, which manifests as a couple’s inability to conceive within a specific time frame.

The prognostic value of semen characteristics, such as sperm count, motility, and concentration as surrogate markers of male fertility, is also confounded by various factors. The fertility potential of a man is influenced by factors such as sexual activity, the functioning of accessory sex glands, and other individual-specific characteristics. Another well-studied marker is the sperm DNA fragmentation index (DFI), which reflects sperm DNA integrity. Though debatable, DFI was shown to be associated with spontaneous and assisted reproductive technology pregnancies and miscarriage [21–23] and recently shown to increase with time in the individual level [24]. Therefore, relying solely on standard semen parameters to predict male fertility outcomes can be challenging and may not provide a comprehensive understanding of the underlying factors contributing to infertility.

This study is subject to several limitations, primarily due to its retrospective design. A prospective longitudinal study with repeated semen samples provided by each patient at regular intervals would have allowed for continuous observation over longer periods and account for surgical interventions with possible effect on sperm quality, such as varicocele repair [25]. Moreover, it would allow to repeat the final semen analysis for accuracy and by that account for the short-term variability in semen parameters. Additionally, the retrospective nature of the study limits the availability of data on the indication for the last SA and for lifestyle, behavioral, nutritional factor modifications, and abstinence period for both first and last semen samples, all of which can potentially impact sperm quality [26], especially BMI, which tends to increase with age and has been negatively correlated with sperm parameters [27]. Although the influence of these factors may be relatively minor at the individual level during a short time duration, their cumulative effects may have significant implications at the population and generational levels.

In conclusion, our findings indicate that there is a decline in sperm parameters over time in the individual level with normal baseline SA evaluated for couple’s infertility, with association to the time element. While a significant percentage of men with normal BSA may experience a decrease in sperm counts below normal clinical reference ranges with time, the direct clinical implications on male fertility remain uncertain. Future research should focus on prospective, longitudinal studies that examine intra-individual changes in sperm quality over clinically significant time periods and allow to account for short-term variability in sperm parameters and alterations in environmental exposures and lifestyle changes. By conducting such studies, clinicians will be able to provide guidance to patients regarding family planning and potentially reduce the need for fertility interventions in the future.

### Supplementary Information

Below is the link to the electronic supplementary material.

Supplementary file1 (TIF 582 KB)

Supplementary file2 (DOCX 14 KB)

## Data Availability

The datasets used and/or analyzed during the current study are available from the corresponding author on reasonable request.
