# Supplementary figures and images for: Changes in Sperm Parameters with Time in Men with Normal and Abnormal Baseline Semen Analysis
Source: Reprod Sci. 2024 Feb 29;31(6):1712–8. doi: 10.1007/s43032-024-01475-1 (PMC11111536; doi:10.1007/s43032-024-01475-1)

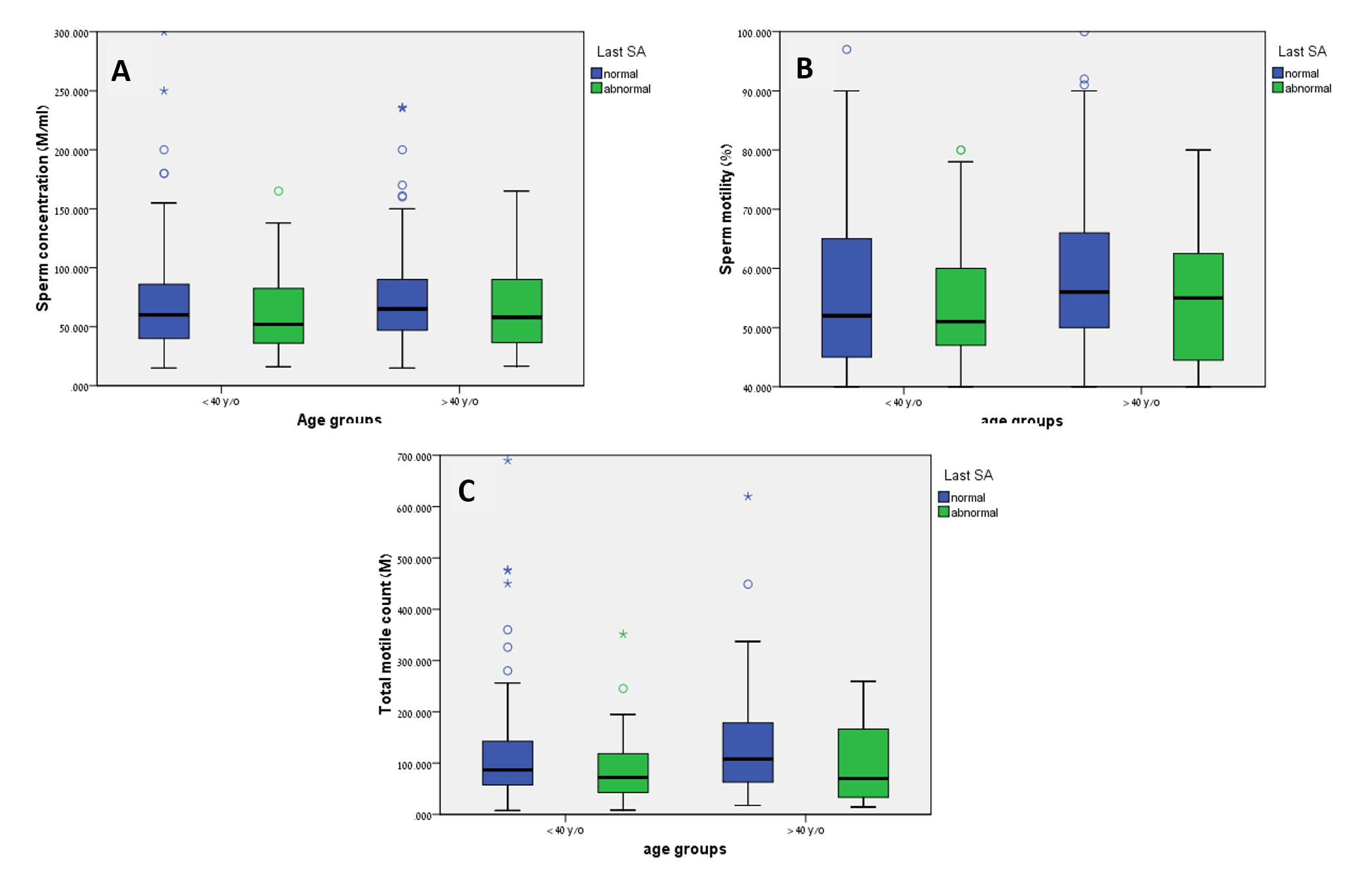

Supplement: Supplementary file 1 — Supplementary file1 (TIF 582 KB) [file 43032_2024_1475_MOESM1_ESM.tif]
