# Supplementary material for: Changes in Sperm Parameters with Time in Men with Normal and Abnormal Baseline Semen Analysis
Source: Reprod Sci. 2024 Feb 29;31(6):1712–8. doi: 10.1007/s43032-024-01475-1 (PMC11111536; doi:10.1007/s43032-024-01475-1)
Supplement: Supplementary file 2 — Supplementary file2 (DOCX 14 KB) [file 43032_2024_1475_MOESM2_ESM.docx]

| **Suppl. Table 1.** Intrapersonal changes with time according to the last semen analysis among individuals with normal baseline semen analysis | | | | |
| --- | --- | --- | --- | --- |
|  | **Normal LSA** | | **Abnormal LSA** | |
| **Parameter** | Mean±SD | p- value* | Mean±SD | p- value* |
| **Change in Volume (ml)** | -0.3±3.7 | 0.296 | -0.1±1.6 | 0.457 |
| **Change in Concentration (M/ml)** | -0.4±53.0 | 0.921 | -19.4±46.3 | <0.001 |
| **Change in motility (%)** | 3.6±17.6 | 0.015 | -22.6±21.3 | <0.001 |
| **Change in TMC (M)** | 3.9±149.8 | 0.766 | -53.7±67.6 | <0.001 |
| **Change in sperm count (M)** | -14.9±227.5 | 0.348 | -42.7±152.0 | 0.004 |
| Data presented as mean ± standard deviation (SD). Note: LSA, last semen analysis.  *Tested using paired t-test. | | | | |
